# Tracing all patients who received insured dialysis treatment in Japan and the present situation of their number of deaths

**DOI:** 10.1007/s10157-021-02163-z

**Published:** 2022-01-01

**Authors:** Shinichiro Kubo, Tatsuya Noda, Tomoya Myojin, Yuichi Nishioka, Saho Kanno, Tsuneyuki Higashino, Masatoshi Nishimoto, Masahiro Eriguchi, Kenichi Samejima, Kazuhiko Tsuruya, Tomoaki Imamura

**Affiliations:** 1 Department of Public Health, Health Management and Policy, Nara Medical University, 840 Shijo-Cho, Kashihara, Nara 634-8521 Japan; 2 Management Innovation Division, Mitsubishi Research Institute, Inc, 10-3, Nagatacho 2-Chome, Chiyoda-Ku, Tokyo, 100-8141 Japan; 3 Department of Nephrology, Nara Medical University, 840 Shijo-Cho, Kashihara, Nara 634-8521 Japan

**Keywords:** Chronic renal insufficiency, Peritoneal dialysis, Administrative claims database, NDB, National database

## Abstract

**Background:**

The survival rate of chronic dialysis patients in Japan remains the highest worldwide, so there is value in presenting Japan’s situation internationally. We examined whether aggregate figures on dialysis patients in the National Database of Health Insurance Claims and Special Health Checkups of Japan (NDB), which contains data on insured procedures of approximately 100 million Japanese residents, complement corresponding figures in the Japanese Society for Dialysis Therapy Renal Data Registry (JRDR).

**Methods:**

Subjects were patients with medical fee points for dialysis recorded in the NDB during 2014–2018. We analyzed annual numbers of dialysis cases, newly initiated dialysis cases– and deaths.

**Results:**

Compared with the JRDR, the NDB had about 6–7% fewer dialysis cases but a similar number of newly initiated dialysis cases. In the NDB, the number of deaths was about 6–10% lower, and the number of hemodialysis cases was lower, while that of peritoneal dialysis cases was higher. The cumulative survival rate at dialysis initiation was approximately 6 percentage points lower in the NDB than in the JRDR, indicating that some patients die at dialysis initiation. Cumulative survival rate by age group was roughly the same between the NDB and JRDR in both sexes.

**Conclusion:**

The use of the NDB enabled us to aggregate data of dialysis patients. With the definition of dialysis patients used in this study, analyses of concomitant medications, comorbidities, surgeries, and therapies will become possible, which will be useful in many future studies.

**Supplementary Information:**

The online version contains supplementary material available at 10.1007/s10157-021-02163-z.

## Introduction

The survival rate of chronic dialysis patients remains higher in Japan than in the United States and Europe [1–3]. This reflects the high level of dialysis treatment in the country, and the benefit of its universal health coverage system. Thus, there is value in presenting Japan’s situation to the world.

In Japan, the Japanese Society for Dialysis Therapy (JSDT) manages the JSDT Renal Data Registry (JRDR), which is a large-scale dialysis statistics registry based on an annual nationwide survey covering various aspects, such as the numbers of patients who underwent hemodialysis (HD), hemodiafiltration, hemofiltration, hemoadsorption, and peritoneal dialysis (PD), as of December 31 each year. Also covered in the registry are the number of newly introduced dialysis cases and the number of deaths among dialysis patients. Because frontline physicians participated in this survey, the aggregate data in the JRDR are considered almost completely accurate, albeit with some selection biases [4, 5]. Given the high response rate of the medical facility questionnaires (98.7% in 2018), the registry is considered the annual dialysis census in Japan.

However, physicians expend a great deal of time and effort to extract necessary data of individual patients to complete the JSDT questionnaire, so a simpler alternative is awaited. The only possible alternative is the National Database of Health Insurance Claims and Special Health Checkups of Japan (NDB) [6]. The NDB is a comprehensive database [7] containing all claims for insured treatment in Japan [8], so it is a complete enumeration of claims for medical treatment provided to individuals insured by Japan’s national health insurance system [9]. It is one of the world’s largest health-related databases [10, 11] containing complete datasets of insured treatments. By using the NDB dataset, selection bias can be reduced and thus valid aggregate figures can be obtained. Also, it is more cost effective to use than nationwide surveys.

We compared these estimates with aggregate data from the JRDR to evaluate whether the NDB can complement a part of the JRDR.

## Materials and methods

### Ethical issues and study design

This descriptive epidemiological study was approved by the Ethics Committee of Nara Medical University (Nos. 1123, 2831). Selected anonymized data were used, so we did not need to explain the study to patients or obtain informed consent.

### Data source

Japan has a system of universal health coverage, and the NDB contains datasets of 138 million individuals (over a 5-year period), regardless of their type of health insurance [12]. The NDB includes data on patients’ personal identification information, month of issue, age group, sex, explanation of procedures implemented, diagnostic code according to the International Classification of Disease (ICD-10, medical care provided, health checkups performed, prescribed medications, and monthly amount claimed. Information about prescribed medications includes brand name, generic name, dosage, and days of medication supply. In this study, we used datasets in this nationwide database and compared them with corresponding data from the JRDR. The data from the JRDR were obtained from the JSDT website [13].

We estimated the annual number of dialysis cases, the annual number of newly initiated dialysis cases, the number of patients on dialysis for these 5 years, treatment situation by age, and the number of deaths among dialysis patients.

### Study population

The JRDR contains data obtained as of the end of each year and annual figures by calendar year, and NDB data from January 2014 to December 2018 were used for aggregation. This was a retrospective cohort study.

### Definition of dialysis patient

Dialysis patients were defined as patients with claims for reimbursement of any “artificial kidney” procedure. Patients who initiated dialysis were defined as those with “additional points for artificial kidney (initiation phase)” in their claims. Dialysis was classified into HD and PD. Procedure codes that define dialysis patients are shown in Online Resource 1. The number of patients who newly initiated dialysis was aggregated annually. Additional medical fee points for dialysis initiation are applicable for a month, so claims linked to 1 case of dialysis initiation can appear in 2 consecutive years. Therefore, to avoid aggregating such duplicates, we prioritized the December claim over a corresponding January claim in the following year. The subjects from the JRDR were defined as (A) all patients who were undergoing chronic dialysis treatment at medical facilities within Japan as of December 31 each year; (B) all patients who newly initiated dialysis in a given year; and (C) patients who received dialysis but withdrew in a given year for reasons, such as death and transplantation. We aggregated NDB data in a manner similar to that used for the JRDR data. We excluded patients who withdrew from dialysis due to acute kidney injury within 2 months. Thus, in the NDB, patients with fee points for dialysis for 2 consecutive months or less were excluded.

Patients who received a combination of PD and HD were counted as PD patients in the JRDR, while they were counted twice in the NDB.

### Definition of death

In the NDB, there is a section to fill in outcome. Death was defined as death recorded as of the annual observation with the presence of medical fee points for dialysis in claims. Thus, withdrawal from dialysis and changes in insurance type were not considered. Patients who initiated dialysis in November 2018 or earlier were not included in this aggregation.

Cases of death in the JRDR, with “death” recorded in the outcome section of the patient report, were aggregated. When patients die after being transferred from one facility to another, the relevant information is expected to be recorded in their report kept in the initial facility as much as possible.

The survival rate was calculated and patients with medical fee points for dialysis in December 2018 were traced for up to 5 years.

## Results

### Total number of dialysis patients and number of patients on each type of dialysis

The total number of dialysis patients was 6–7% lower in the NDB than in the JRDR, and the number of patients who newly initiated dialysis was almost identical (Table 1). Numbers of patients on each type of dialysis are shown alongside the total numbers of dialysis patients each year in Table 2. The number of HD patients was lower in the NDB than in the JRDR, while the number of PD patients was higher in the NDB.

**Table 1:** Comparison of the total number of dialysis patients

	Number of patients (% versus reference)			
Year	Dialysis (as of the end of year)		Initiation of dialysis (annual)	
	NDB	JRDR	NDB	JRDR
2014	300,069	320,448	37,848	38,327
	(93.6%)	(reference)	(98.8%)	(reference)
2015	305,878	324,986	39,136	39,462
	(94.1%)	(reference)	(99.2%)	(reference)
2016	309,700	329,609	39,408	39,344
	(94%)	(reference)	(100.2%)	(reference)
2017	312,955	334,505	40,815	40,959
	(93.6%)	(reference)	(99.6%)	(reference)
2018	313,031	339,841	39,370	40,468
	(92.1%)	(reference)	(97.3%)	(reference)

*JRDR* Japanese Society for Dialysis Therapy renal data registry, *NDB* National database of Health Insurance Claims and Special Health Checkups of Japan

**Table 2:** Comparisons of the total numbers of dialysis patients, and patients on different types of dialysis

Year	Dialysis (overall)		HD		PD	
	NDB	JRDR	NDB	JRDR	NDB	JRDR
2014	300,069	320,448	293,164	298,924	9,828	9,255
	(93.6%)	(reference)	(98.1%)	(reference)	(106.2%)	(reference)
2015	305,878	324,986	299,039	302,501	9,856	9,322
	(94.1%)	(reference)	(98.9%)	(reference)	(105.7%)	(reference)
2016	309,700	329,609	302,960	308,503	9,706	9,021
	(94%)	(reference)	(98.2%)	(reference)	(107.6%)	(reference)
2017	312,955	334,505	305,971	310,708	9,610	9,090
	(93.6%)	(reference)	(98.5%)	(reference)	(105.7%)	(reference)
2018	313,031	339,841	305,498	316,113	9,618	9,445
	(92.1%)	(reference)	(96.6%)	(reference)	(101.8%)	(reference)

Note: In the NDB, HD patients include those who underwent HD and PD

*HD* hemodialysis, *JRDR* Japanese Society for Dialysis Therapy renal data registry, *NDB* National database of Health Insurance Claims and Special Health Checkups of Japan, *PD* peritoneal dialysis

### Tracing of dialysis patients and verification of death cases

Table 3 shows the length of dialysis therapy (up to 5 years) among patients who received dialysis in December 2018. In the JRDR, only data from a 5-year period were aggregated, so data in the previous 1–4 years are missing in the table. When comparing NDB and JRDR, differences in the proportions of men and women who received dialysis for 5 years were 1–2%, and the proportions in 2018 were the same in both databases.

**Table 3:** Back-tracing the number and percentage of patients on continuous dialysis care in the 5 years preceding 2018 as the reference year

		End of 2018	1 year earlier	2 years earlier	3 years earlier	4 years earlier	5 years earlier
NDB	Men	206,089	179,230	155,665	135,016	116,659	100,093
		(100%)	(87.0%)	(75.5%)	(65.5%)	(56.6%)	(48.6%)
	Women	110,776	98,730	87,816	78,163	69,125	60,787
		(100%)	(89.1%)	(79.3%)	(70.6%)	(62.4%)	(54.9%)
JRDR	Men	213,881					107,577
		(100%)					(50.3%)
	Women	113,173					64,110
		(100%)					(56.6%)

*JRDR* Japanese Society for Dialysis Therapy renal data registry, *NDB* National database of Health Insurance Claims and Special Health Checkups of Japan

**Fig. 1:**
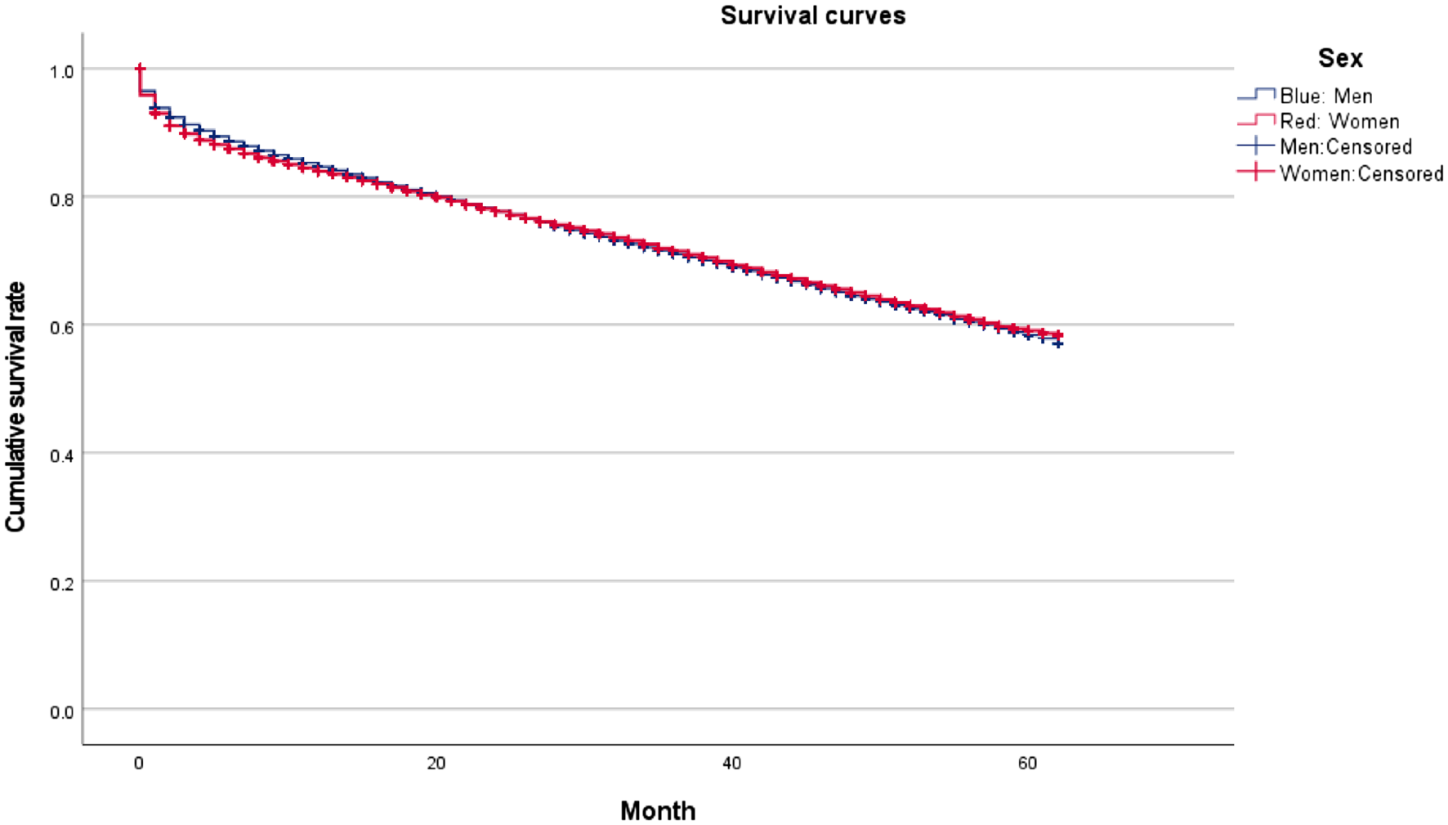
Five-year survival curves of patients who initiated dialysis between January 2014 and December 2014

Figure 1 shows 5-year survival curves who initiated dialysis during January 2014–December 2014. The survival rate at dialysis initiation was 6 percentage points lower in the NDB than in the JRDR, indicating that the NDB includes more patients who die soon after dialysis initiation.

To examine the data in more detail, we summarized 4-year cumulative survival rates of patients who initiated dialysis in 2014 by age group in Table 4. To comply with the data disclosure rules of the NDB, exact patient numbers are not shown in some parts of the table, and 4-year cumulative survival rates of patients aged 2–14 years and 15–29 years are not reported here. Cumulative survival rates were roughly the same between the NDB and JRDR, though some patient numbers differed.

**Table 4:** Four-year survival rate of patients who initiated dialysis in 2014 by age group

		NDB			JRDR		
		Men	Women	Total	Men	Women	Total
Age 0–14	No. of patients	< 10	< 20	21	10	15	25
	Four-year cumulative survival rate	–	–	–	0.68	0.91	0.83
Age 15–29	No. of patients	70–79	40–49	115	131	76	207
	Four-year cumulative survival rate	–	–	–	0.97	0.91	0.95
Age 30–44	No. of patients	571	199	770	1,281	478	1,759
	Four-year cumulative survival rate	0.95	0.95	0.95	0.94	0.95	0.94
Age 45–59	No. of patients	2,774	1,044	3,818	4,021	1,500	5,521
	Four-year cumulative survival rate	0.90	0.91	0.90	0.89	0.91	0.90
Age 60–74	No. of patients	7,814	3,229	11,043	9,762	4,076	13,838
	Four-year cumulative survival rate	0.76	0.81	0.78	0.77	0.82	0.78
Age 75–89	No. of patients	10,360	5,516	15,876	8,321	4,995	13,316
	Four-year cumulative survival rate	0.57	0.61	0.59	0.56	0.59	0.57
Age 90 +	No. of patients	950	931	1,881	347	352	699
	Four-year cumulative survival rate	0.49	0.52	0.50	0.34	0.40	0.37
Not recorded	No. of patients				1	2	3
	Four-year cumulative survival rate				0.00	0.00	0.00
Total	No. of patients	22,549	10,975	33,524	23,874	11,494	35,368
	Four-year cumulative survival rate	0.69	0.70	0.69	0.72	0.72	0.72

*JRDR* Japanese Society for Dialysis Therapy renal data registry, *NDB* National database of Health Insurance Claims and Special Health Checkups of Japan

The annual number of deaths among dialysis patients is shown in Table 5. The ratio of the number of deaths in the JRDR to that in the NDB was around 93% each year. Thus, the number of deaths tended to be 7% lower in the NDB than in the JRDR, similar to the number of patients.

**Table 5:** Annual number of deaths among dialysis patients

	2014	2015	2016	2017	2018
NDB	28,218	29,129	30,236	31,024	31,300
	(91.9%)	(93.8%)	(95.1%)	(95.4%)	(92.4%)
JRDR	30,707	31,068	31,790	32,532	33,863
	(reference)	(reference)	(reference)	(reference)	(reference)

*JRDR* Japanese Society for Dialysis Therapy renal data registry, *NDB* National database of Health Insurance Claims and Special Health Checkups of Japan

## Discussion

### Definition of dialysis patients and comparison of the number of patients

The number of dialysis patients was 7% lower in the NDB than in the JRDR. The NDB includes data about insured treatment only and does not include information about patients who completely rely on public assistance, meaning that the number of dialysis patients is likely to be underestimated. According to a government survey, public assistance recipients totaled 2.09 million in February 2019 [14], accounting for 1.6% of the population. Meanwhile, among patients who applied for medical expense grants for intractable disease treatment, public assistance recipients totaled 2.3% (20,930/892,123) [15]; the actual figure could be higher because some patients with an intractable disease did not apply for the grant if they were already living on social welfare benefits [16]. The proportion is likely to be higher than 2.3% if patients are receiving continuous treatment, and there is uncertainty about whether this figure can be applied to dialysis patients. However, this problem will be solved in the future because data related to public assistance recipients will become available from the NDB for aggregation after amendment of the system.

Also, the NDB does not contain data of a very small population of untraceable patients, likely because claims were not processed electronically due to high medical expenses (transplantation, etc.). The effect on the population of dialysis patients is unknown but the inclusion of such untraceable cases needs to be taken into consideration.

Meanwhile, most of the data were entered manually in the JRDR, which can be either overestimation or underestimation. As example of overestimation, some patients who were hospitalized at the time of the statistical survey might have been doubly counted at the outpatient facility they had visited previously. Also, because many patients are over 100 years old, patients who have died may be treated as alive. Factors contributing to the underestimation include the possibility that a small number of facilities not belonging to JSDT are not included, and that data are not entered due to ambiguity in the primary entry facility.

Aggregate numbers of patients who initiated dialysis were almost the same in the NDB and the JRDR, although that in the NDB was slightly lower than that in the JRDR. This similarity might be due to some patients being insured at initiation of dialysis but then becoming exempt as their situation changed, for example, patients who begin to receive public assistance due to their inability to work.

The number of HD patients was lower in the NDB than in the JRDR, while that of PD patients was higher in the NDB. Patients who received a combination of PD and HD were counted twice in the NDB, whereas they were counted as PD patients in the JRDR. It should be noted that the number of patients might be lower than the actual number because claims for fee points for either HD or PD are accepted within a month. Some patients receive home PD, which might have contributed to the high number of PD patients in the NDB. Home PD patients usually make monthly visits to clinics and/or hospitals, but some opt for home-based medicine because outpatient visits are difficult. Dialysis treatments in such cases might not be reflected in the JRDR as they were not performed at medical facilities. However, this is a limited case. Also, completely switching from PD to HD can be handled as the combination of PD and HD depending on the timing of data aggregation, and this might also have contributed to the high number of PD cases. Further, some dialysis treatments (especially PD treatments) might have been performed at medical facilities that did not participate in the JRDR but were included in the NDB. There was a report on a technique that uses several algorithms to identify patients receiving PD by using health insurance claims data in the United States, but it did not compare findings with data from other sources [17].

### Tracing the history of dialysis and deaths

The situation of dialysis over a 5-year period prior to the reference observation point was similar in the NDB and JRDR, regardless of sex. Data in the NDB can be traced back to only around 10 years, even when including data not considered in this study, whereas data in the JRDR can be traced back 40 years. If data continue to accumulate in the NDB, then a range of data aggregation will become available.

Survival curves of patients who received dialysis between January 2014 and December 2018 showed a 5-year survival rate of roughly 60%, which is in good agreement with the results in the JRDR (i.e., 66.1% in 2018).

Including patients who received dialysis for 2 months or less in the survival curves revealed that slightly less than 10% of patients died soon after dialysis initiation. This finding is probably because the condition of patients is most severe and unexpected complications are most likely to occur around the dialysis initiation. Patients often present with unstable blood pressure due to sepsis or myocardial infarction soon after initiation of dialysis. Such a poor condition can lead to impaired renal function, requiring temporary initiation of dialysis. For example, dialysis initiation for rapid progressive glomerulonephritis due to microscopic polyangiitis often results in death due to infection. Dialysis treatment for acute renal failure was excluded if it was continued for 2 months or less in the JRDR, so there might be agreement in survival rate between the NDB and in the JRDR if the same conditions are used.

Cumulative survival rates by age group in each sex in the NDB showed similar trends to those in the JRDR, although precise comparison is difficult. One of the reasons is a difference in aggregation timing: age was determined as of the end of year in the NDB, so there can be a maximum difference of 3 months between the NDB and the JRDR. The patient questionnaire for collection of JRDR data had a response rate of 94.7%, which may have affected the figures. Also, cases of withdrawal of dialysis for reasons, such as kidney transplantation, are not taken into account in the NDB, meaning that the cumulative survival rates obtained here might differ from the actual figures.

The annual number of deaths was 10% lower in the NDB than in the JRDR. Possible reasons for this include missing or incorrect data input in the NDB due to errors at medical facilities, changes in insurance type because patients began receiving public assistance, and temporary omissions due to participation in clinical studies.

### Summary of issues and future usability

Key aggregate figures obtained in this study are summarized in Online Resource 2 to clarify issues to be addressed. Collectively, the aggregate figures related to dialysis obtained from the NDB closely corresponded to figures from the JRDR, even though the data sources are different. Generally, in descriptive statistics, when there are no substantial discrepancies in aggregate figures between two very different aggregation methods, it can be inferred that neither aggregate figure is the true figure, but the true figure is likely to be close to the figures estimated by those methods. Because the data aggregation methods were very different between the NDB and the JRDR, this inference is applicable.

There are biases in both databases. There is underestimation in the NDB due to the non-inclusion of data of public assistance recipients and the use of a different counting method, overestimation in the JRDR due to duplication of patients upon transfer between medical institutions, and underestimation in the JRDR due to the non-inclusion of patients who received treatments at medical facilities that do not belong to JSDT. The JRDR is built upon the enormous efforts of frontline medical professionals, which imposes a considerable burden. Automated data sampling is used in some facilities, but its wider spread is awaited. Replacing some items in the JRDR with the relevant items in the NDB would enable data to be obtained more quickly and easily.

As previously reported, research papers using health insurance claims data should provide validation for the validity of diagnosis codes [18], and thus, the definition of patients is highly important [19, 20]. However, detailed aggregation of data related to dialysis patients is not possible with the NDB, so these databases must be selected depending on the purpose of use.

This study showed that the aggregation of dialysis patient data in the NDB is valid because it closely corresponds to aggregation of the corresponding data in the JRDR. This indicates the possibility of performing more detailed analyses (e.g., follow-up studies using concomitant medications and incidence of comorbidities as outcomes, and studies using dialysis as an exposure). Tracing the medical history before dialysis initiation is difficult using the JRDR. The present preliminary study will likely serve as a basis for many studies that use the definitions in this study.

The reasons why prognosis in dialysis patients is more favorable in Japan than overseas remain unclear [21]. Possible reasons include a lower rate of catheter use (a higher rate of arteriovenous fistula use) for vascular access [22] and longer dialysis sessions [23] in Japan. Duration of dialysis sessions in Japan is longer than that in the US, but similar to that in Europe, and shorter than that in Australia. It is noteworthy, however, that the survival rate increases as the duration of dialysis sessions increases in Japan [24]. Information, such as concomitant medications, incidence of comorbidities, and clinical course, will be useful in other countries.

### Limitations and recommendation

Data were aggregated for each month, so a switch from HD to PD and differences due to the combination therapy were not taken into account. Test results are not available in the NDB, so it is not possible to use the definition of patients used in the diagnostic guidelines for dialysis initiation [25].

## Conclusion

Using the NDB can simplify data aggregation compared with using the JRDR, so the NDB can be considered a source of new indices. The NDB is the largest medical-related cohort worldwide and contains many indexes, and aggregation of its data will provide valuable knowledge on many other diseases. This will increase the utility value of the NDB, and the definitions used for dialysis patients in this study will be important for future studies.

## Supplementary Information

Below is the link to the electronic supplementary material.

Supplementary file1 (XLSX 15 KB)
